# Coping with burns: the role of coping self-efficacy in the recovery from traumatic stress following burn injuries

**DOI:** 10.1007/s10865-015-9638-1

**Published:** 2015-04-08

**Authors:** Mark W. G. Bosmans, Helma W. Hofland, Alette E. De Jong, Nancy E. Van Loey

**Affiliations:** INTERVICT, Tilburg University, Tilburg, The Netherlands; Maasstad Hospital, Rotterdam, The Netherlands; Association of Dutch Burns Centers, Rotterdam, The Netherlands; Red Cross Hospital, Beverwijk, The Netherlands; Association of Dutch Burns Centers, Beverwijk, The Netherlands; Department of Clinical and Health Psychology, Utrecht University, Utrecht, The Netherlands

**Keywords:** Coping self-efficacy, Posttraumatic stress, Burn patients, Quality of life, Coping, Latent growth curve modeling

## Abstract

We conducted a three-wave prospective study among patients with burns (N = 178) to examine the prospective influence of coping self-efficacy (CSE) perceptions on trajectories of posttraumatic stress symptoms in the first 12 months after burn injuries. Using linear growth curve modeling, we corrected for demographics, the number of surgeries during initial admittance, trait coping styles, and changing levels of health-related quality of life. CSE during initial admission was by far the strongest predictor of both initial PTSD symptoms and degree of symptom change with higher CSE levels associated with lower initial symptoms and a steeper decline of symptoms over time. Of the other variables only avoidant coping was associated with higher initial symptom levels, and only emotional expression associated with greater rate of recovery. Current findings suggest that CSE plays a pivotal role in recovery from posttraumatic stress after a burn injury, even when the role of burn-related impairments is taken into consideration. Implications of findings are discussed.

## Introduction

Burn survivors are at risk to suffer from severe long-term psychological problems (Fauerbach et al., 2007; McKibben et al., 2009). Symptoms of acute stress disorder (ASD), posttraumatic stress disorder (PTSD), anxiety, depression, as well as delirium and problems with sleeping and frequent nightmares are commonly experienced in the aftermath of severe burn injuries (Davydow et al., 2009; Thombs et al., 2006). However, as observed after other potentially traumatic events, PTSD prevalence tends to decrease over time among burn survivors. Around 10 % continue to suffer from chronic PTSD and about 15 % from sub-threshold symptom levels after 12 months postburn (Dyster-Aas et al., 2008; Van Loey et al., 2008). Despite extensive research into risk- and protective factors associated with post-burn mental health (Sareen et al., 2013), it is still not completely understood why many survivors will recover from clinically relevant stress levels whereas others will maintain high traumatic stress symptom levels, and it remains difficult to predict who will exhibit which pattern of psychological recovery over time.

Coping self-efficacy (CSE), the perceived capability to effectively deal with posttrauma recovery demands, has been shown to have a strong protective effect in other trauma populations (Luszczynska et al., 2009). CSE perceptions have been found to positively influence both immediate and long-term stress levels after exposure to very diverse traumatic events such as disasters, terrorist attacks, motor vehicle accidents, combat and domestic violence. In longitudinal studies, CSE perceptions accounted for 8–27 % of the variation in PTSD symptoms over and above the effect of previous symptom levels (e.g. Benight et al., 2004; Bosmans et al., 2013; Luszczynska et al., 2009).

CSE affects the stressfulness of traumatic events in three ways. First, CSE perceptions affect the degree to which an event is perceived as threatening resulting from the perceived balance between coping abilities, coping demands, and the potential harmfulness of the event (Bandura, 1997). Second, CSE perceptions may influence the motivation to employ coping strategies as well as the type of strategies that are considered because of its influence on the expected outcomes of behavior (Bandura et al., 1969; Bandura et al., 1985). And third, CSE affects the degree to which (initial) PTSD symptoms are perceived as stressful; it determines the perception of control over disturbing thoughts and emotions (Kent, 1987; Kent & Gibbons, 1987). CSE can also be seen as the essential step in Lazarus and Folkman’s transactional model of stress and coping (Lazarus & Folkman, 1984): it determines secondary appraisal (evaluation of coping options) and thereby the actual coping efforts employed, since the only viable coping options are those that the individual perceives as within ones capabilities. Previous research has shown that CSE has a positive influence on the use of effective coping strategies (Benight et al., 1999b). In sum, CSE reflects the perceived level of capability to effectively deal with the event and its consequences, and determines appraisal of the event and its consequences.

Appraisals of the trauma and its consequences play a central role in the cognitive model of PTSD developed by Ehlers and Clark (2000). According to this model, individuals with PTSD have appraisals that create a sense of current threat, either external (e.g. the world as a dangerous place) or internal (e.g. views of one’s self as incompetent or unworthy). The perceived threat induces cognitive and behavioral responses that can be either adaptive or maladaptive. Whereas repeated emotional expression may facilitate the processing of the event by habituation and reduction of perceived threat, avoidance may be a strategy that is helpful on the short term but interferes with processing of the event and therefore prevents change (Ehlers and Clark, 2000; Ehlers et al., 2006). Previous research among burn victims has shown that the coping style avoidant coping was associated with worse (mental) health outcomes, while active coping and seeking social support were associated with better (mental) health outcomes (Amoyal et al., 2011; Bryant, 1996; Kildal et al., 2005; Lawrence & Fauerbach, 2003; Ptacek et al., 1995; Willebrand et al., 2004).

Besides the psychological impact, patients with burns are typically faced with physical trauma that has been shown to affect long-term functioning. The physical problems patients with burns are confronted with can be significant and form an important factor across the whole recovery process. Burn events can be psychologically traumatic because of life threat, horrible images at the scene, and witnessing large skin damage. During the acute phase painful wound dressing changes and painful physiotherapeutic exercises can place a heavy toll on the patient. But also after hospitalization the scars continue to challenge the psychological and physical recovery process in which the patients has to integrate body image changes, and deal with functional impairments, as well as endure painful procedures to prevent tissue contractures and optimize functioning (Esselman et al., 2006; Fauerbach et al., 2002; Summer et al., 2007; Thombs et al., 2008). Furthermore, burn survivors may suffer from chronic pain (Schneider et al., 2006), which is often comorbid with PTSD symptoms (Asmundson et al., 2002). This association may be due to shared vulnerability for development of pain and PTSD, or due to mutual maintenance, whereby pain and PTSD exacerbate each other (Asmundson et al., 2002).

A composite measure of physical and psychological health is health related quality of life (HRQOL). Overall, burn survivors have lower levels of HRQOL and higher levels of emotional distress than the general population (Stavrou et al., 2014). Despite lower average HRQOL levels however, there is evidence for satisfactory levels among burn survivors including among those with severe injuries; in most but not all domains they return to norm levels (Anzarut et al., 2005; Stavrou et al., 2014; Van Loey et al., 2012). Of note, HRQOL trajectories have been found to be negatively influenced by acute PTSD/traumatic stress symptoms following a burn event (Fauerbach et al., 1999; Renneberg et al., 2014; Van Loey et al., 2012). Moreover, while there is significant improvement in HRQOL over time, patients reporting higher initial levels of PTSD symptoms showed a significantly lower improvement over an 18-month period (van Loey et al., 2012). Both the psychological and physical problems, including pain, are important components of HRQOL and may affect CSE in the aftermath of a burn injury.

Previous studies have demonstrated the central role of CSE in recovery from trauma. At this time however, it is unknown whether CSE is also important in psychological recovery among burn survivors who may have to deal with long-term functional problems. A relevant question arises as to whether CSE assessed in the acute phase after the burn event where the healing process takes a heavy toll, would be predictive for the recovery from traumatic stress symptoms. The feeling of control which is central to CSE may be influenced by the physical state shortly after the burn event. Nevertheless, the early identification of individuals that are able to cope with the trauma is of clinical importance as they may need a different kind of support during recovery.

The primary aim of this study was to examine the influence of CSE perceptions on trajectories of PTSD in the first 12 months after burn injuries taking into account the changing level of HRQOL. We hypothesized that CSE contributes independently on the course of PTSD symptoms among burn survivors. For this purpose we conducted a three-wave prospective study among patients with burns admitted to burn centers in the Netherlands and Belgium. We corrected for demographics, the number of surgeries needed during initial admittance, trait coping styles and changing HRQOL levels. Unique in this study is that we examined not only PTSD trajectories, but also take into account the effect changes in HRQOL have on PTSD symptom levels over time.

## Methods

### Sampling and procedure

The results of this study are part of a larger study among patients with burns in the Netherlands and Belgium. The study included 215 patients who were admitted to one of five burn centers in the Netherlands or in Belgium between May 2010 and September 2012. A total of 339 patients met the inclusion criteria of which 84 declined participation and 40 could not be invited according to the study schedule and 215 signed informed consent (63 %); 178 of which provided valid scores on the outcome variable at T1, comprising the final sample (individuals with missing values on the PTSS scale at T1 were excluded). The 124 patients not included did not differ from the 215 included patients on age, gender, and length of stay in hospital but they had a higher total body surface area burned (*t* = −2.599, *df* = 328, *p* = .01). The study was approved by an ethical committee in the Netherlands and in Belgium. Patients were invited to participate into the study by a local researcher. After providing written informed consent patients received printed questionnaires 2–4 weeks (T1), 6 months (T2) and 12 months (T3) after the event.

Of the 178 respondents who provided valid scores on the outcome variable at T1, 48 dropped out. Non-response analyses showed that full respondents and those who dropped out differed on two variables. Dropouts were younger (mean age 36.23 and 42.75 respectively, *t*(176) = −2.53, *p* = .012), and scored lower on the coping trait seeking support (1.96 and 2.15 respectively, *t*(107.88) = −2.046, *p* = .043).

## Measures

### PSTD symptoms

To examine event-related PTSD symptoms at T1, T2 and T3, we used the original 15-item IES (Horowitz et al., 1979) and the 6 hyperarousal items of the Impact of Event Scale-Revised (IES-R, Weiss & Marmar, 1997). The symptom clusters are related to a specific traumatic event. The original scoring system of the IES was used, however. We will call this version of the IES(-R) the IESplus. This approach has been used in previous research (cf Pfefferbaum et al., 2000, 2002, 2003), and has the benefit of comparability with results obtained using the original IES, while still allowing for the measurement of all three symptom clusters of PTSD. The construct validity and reliability of the Dutch version of the IES was proven to be acceptable across different traumatic experiences (Van der Ploeg et al., 2004). Cronbach’s alpha’s for the IESplus total scores in the present sample was high at all waves (.94, .96 and .96 respectively). Scores on the IESplus range from 0 to 105.

### Coping self-efficacy

The 7-item coping self-efficacy measure (CSE-7, Bosmans et al., in press) was administered at T1 to assess trauma-related CSE. This scale is based on a 20-item trauma-related scale developed by (Benight et al., 1999a; Benight et al., 2004). The CSE-7 has a robust factor structure across very different types of PTEs, making it especially suitable for use in populations with mixed trauma exposure (Bosmans et al., 2014). For each item, respondents rated their perceived efficacy on dealing with different consequences of the disaster on a 7-point scale (e.g. ‘resuming normal life’; ‘dealing with frightening images or dreams about the event’; ‘being optimistic since the event’). Possible scores range from 7 (lowest self-efficacy) to 49 (highest self-efficacy). In this study, the internal consistency of the CSE scale was high (.88).

### Health-related quality of life

The Euroqol-5D-3L (Brooks, 1996) was used to assess health-related quality of life at T1, T2 and T3. The Euroqol-5D measures health state on 5 dimensions: mobility, self-care, usual activities, pain/discomfort, and anxiety/depression. Each dimension is rated on a 3-point scale, from no problems to severe problems. A summary score, transforming these five dimensions into one score, was calculated using a scoring algorithm based on empirical valuations from the UK general population. The summary score can range from 1 (full health) to 0 (for death) (Dolan, 1997). The scale showed to be useful in burn populations (Oster et al., 2009).

### Coping styles

The UCL-B (Utrecht Coping List Brief) was used to assess general trait coping styles at T1. This 26-item scale assesses 7 different coping styles: emotional expression, seeking social support, active coping, avoidance coping, palliative reactions, soothing thoughts and wishful thinking, and depressive reactions (Storsbergen, 2004). The scale is a shortened version of the original UCL (Schreurs & van de Willige, 1988). The scale was shown to have sufficient internal consistency and high test–retest reliability (Schreurs et al., 1993). The coping styles which have been shown to be most relevant for recovery after trauma among burn victims (Amoyal et al., 2011; Bryant, 1996; Kildal et al., 2005; Lawrence & Fauerbach, 2003; Ptacek et al., 1995; Willebrand et al., 2004) were included in the analyses: active coping (e.g. coming up with several options to solve a problem), seeking social support (e.g. sharing your concerns with someone), avoidant coping (e.g. avoiding difficult situations) and emotional expression (e.g. letting ones annoyance show). In this study, the internal consistency of most of the subscales were good (active coping: .88, seeking social support: .85, avoidant coping: .67, emotional expression .70).

### Statistical analyses

In order to examine the influence of CSE at admission and changing HRQOL levels on trajectories of PTSD among burn patients in the first 12 months after admission, we used linear growth curve modeling (LGM) with time-invariant and time-varying covariates using Mplus 6.1 (Muthén & Muthén, 2007, p 114–115). A number of consecutive models were estimated. As a first step, the linear growth curve model for PTSD levels (Model 1a) was estimated. In order to determine the shape of the curve (linear or quadratic), a quadratic time term was added (Model 1b). Time points for the slope factor were set at 0, 6 and 12, reflecting a linear growth model with 6 month intervals between measurements. In the next step, the time varying predictors (HRQOL levels) were added (model 2), in effect correcting for the influence of their changing levels. Finally, CSE perceptions during initial admission and the time-invariant covariates were added to the model [Demographics (age, gender), number of surgeries and coping styles]. Only the significant covariates shown in Table 3 have been estimated. Non-relevant and non-significant covariates were constrained to equal 0 for parsimony. For full information on correlations between variables in the model see Table 4. Mplus version 6.1 (Muthén & Muthén, 2007) was used to estimate the models. Maximum Likelihood estimation with Robust standard errors (MLR) estimation was used because of the high number of variables in the model with non-normal distributions. This robust full information maximum likelihood estimator provides a robust χ^2^ test (Kaplan, 2008). Because MLR was used to estimate the models, χ^2^ values reported are Santorra–Bentler scaled (mean-adjusted), where the Chi square statistic is divided by a scaling correction. Since LGM is robust to unequal numbers of observations across time (Chin et al., 2009), cases with missing observations on T2 or T3 remained in the analyses. Model fit was evaluated using the Comparative Fit Index (CFI) the Tucker–Lewis Index (TLI), and the root mean square error of approximation (RMSEA). The criteria for good model fit proposed by Hu and Bentler (1999) were used: CFI and TLI > .90 and RMSEA < .08.

## Results

### Sample characteristics

Descriptives for the sample are shown in Table 1. Average PTSD symptom levels declined over time, while HRQOL levels increased substantially over time, especially between T1 and T2.

**Table 1:** Descriptives

	M/%	SD
Age	40.99	15.48
Gender (male)	66.3 %	
Number of surgeries (0)	.70	.71
CSE	40.38	8.26
Coping: emotional expression	1.78	.70
Coping: seeking social support	2.10	.63
Coping: active	2.64	.67
Coping: avoidant	1.79	.53
HRQOL at T1	.56	.34
HRQOL at T2	.85	.20
HRQOL at T3	.87	.21
PTSD at T1	25.57	23.24
PTSD at T2	21.10	23.46
PTSD at T3	17.00	22.19

### Latent growth curve analyses

The simple latent growth curve model (Model 1a) had good overall fit: χ^2^ (1, N = 178) = .000, *p* = .988, CFI = 1.000, TLI = 1.045, RMSEA = .000 (CI .000–.000). Results indicated that PTSD symptoms decreased over time, with an estimated mean at T1 of 25.56 and a significant decline in symptoms over time (−.682, Z = −4.586, *p* < .001). Additionally, there was significant variance in both the intercept (Di = 399.429, CI 325.093–473.765, *p* < .001) and the slope of PTSD symptoms (Ds = 3.04, CI 2.007–4.073, *p* = .003), indicating individual differences in both initial symptom levels and in change over time. The significant and negative *F*(1, 178) = −12.188, *p* = .056^1^) covariance between the intercept and slope indicates that those who score high on initial PTSD symptom levels tend to have a lesser degree of decline in symptoms over time. The shape of the slope (linear or quadratic) was tested by adding a quadratic slope factor to the model (Model 1b). Results of this model showed that the slope is linear. The quadratic slope factor was therefore not included in Models 2 and 3.

Adding the time-varying HRQOL levels to the model (Model 2) resulted in a model with good fit: χ^2^ (7, N = 178) = 10.702, *p* = .044, CFI = .966, TLI = .927, RMSEA = .077 [CI .013–.134]. Higher levels of HRQOL were associated with lower PTSD symptom levels, with the association becoming stronger with each measurement (T1: *β* = −.25 *Z* = −3.442, *p* = .001; T2: *β* = −.45 *Z* = −3.827, *p* < .001; T3: *β* = −.65 *Z* = −6.461, *p* < .001).

**Fig. 1:**
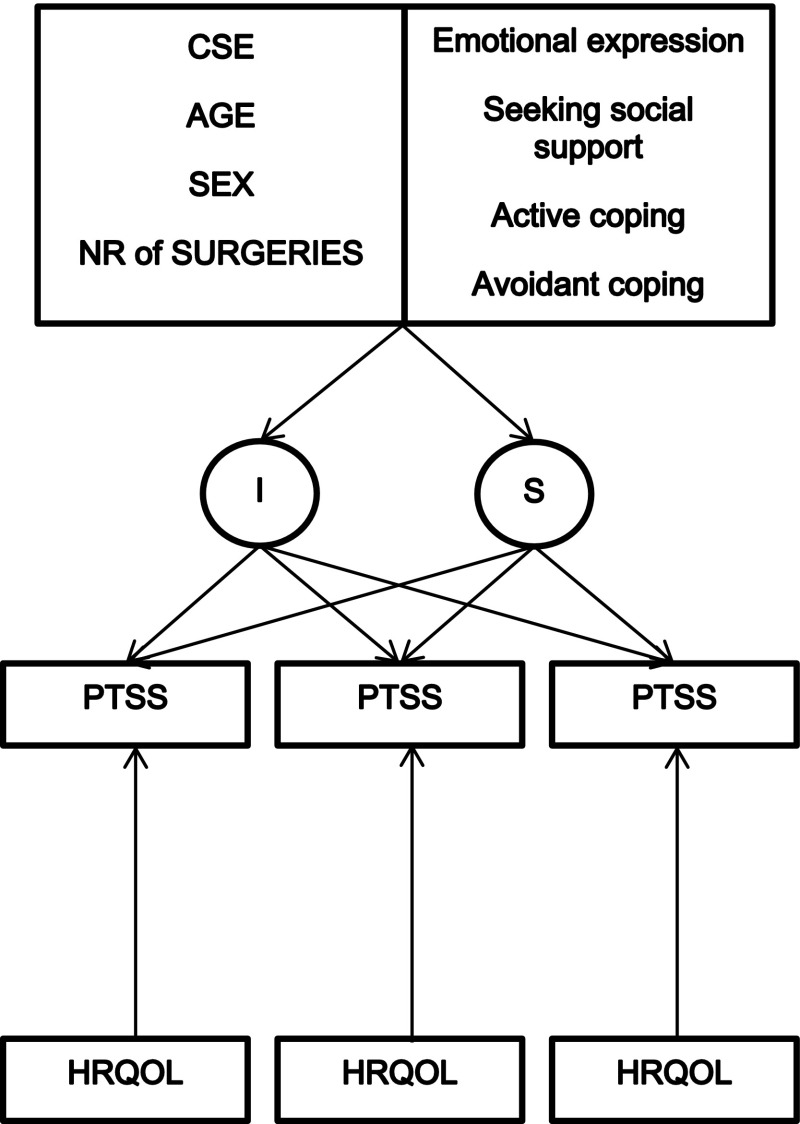
Final model

The final model which included CSE, coping styles, demographics and burn severity measured at T1 (Model 3, see Fig. 1) also had good overall fit χ^2^ (54, N = 178) = 80.238, *p* = .001, CFI = .881, TLI = .921, RMSEA = .062 [CI .039–.083]. Results (see Table 2) show that 26.2 % of variance in individual development of PTSD symptoms was explained in the model. Of the predictors, CSE is negatively associated with initial PTSD symptom levels (*β* = −.67 *Z* = −8.287, *p* < .001), and avoidant coping is positively associated with PTSD levels (*β* = .144 *Z* = 1.940, *p* = .052).^2^ None of the other demographic variables, number of surgeries, nor the remaining coping styles are significantly related to initial PTSD levels. When we look at the development of PTSD symptomatology over time, only CSE (*β* = .46 *Z* = 4.558, *p* < .001) and emotional expressive coping (*β* = .13 *Z* = 2.002, *p* = .045) have a significant impact on the slope of symptoms, with higher levels of CSE and higher levels of emotional expression associated with a greater slope of recovery. In other words, when correcting for demographics, number of surgeries and coping styles, only CSE perceptions and emotional expressive coping independently affect the rate of decline in PTSD symptoms. After adding the time-invariant predictors to the model, HRQOL at T1 is no longer significantly associated with PTSD symptom levels. For covariates in the final model see Table 3.

**Table 2:** Main effects within final model

Model	Estimate	SE	β
Time-varying factors			
HRQOL T1	−.364	4.112	−.005
HRQOL T2***	−25.584	5.450	−.227
HRQOL T3***	−49.974	9.457	−.575
Intercept			
Age	−.125	.086	−.094
Sex^a^	.989	2.950	.023
Surgeries	.029	2.075	.001
Active coping	−.720	2.014	−.023
Avoidant coping*	5.618	2.896	.144
Seeking support	.850	2.324	.026
Emotional expression	2.055	1.701	.070
CSE***	−1.693	.204	−.673
Slope			
Age	.001	.008	.005
Sex^a^	.023	.285	.006
Surgeries	−.198	.222	−.081
Active coping	.062	.173	.024
Avoidant coping	−.067	.270	−.021
Seeking support	−.044	.173	−.016
Emotional expression*	.321	.161	.130
CSE***	.097	.021	.461
Explained variance			
Intercept***	.531	.078	
Slope**	.262	.106	

* *p* < .05; ** *p* < .01; *** *p* < .001

^a^Male is the reference category

**Table 3:** Covariances within final model

	Estimate	SE
HRQOL T2 with		
HRQOL T1**	.010	.004
HRQOL T3***	.035	.011
CSE with		
Surgeries***	−1.264	.390
Avoidant coping*	−.659	.317
Active coping***	1.220	.333
HRQOL T1***	1.033	.188
Active coping with		
Avoidant coping*	−.055	.011
Age***	−.065	.021
Seeking support**	.096	.038
Seeking support		
HRQOL T1**	−.044	.016
Sex**^,a^	.055	.022
Age**	−1.619	.639
Avoidant coping with		
Sex***^,a^	.059	.019
Surgeries with		
Avoidant coping**	.076	.025
Age***	2.460	.801
HRQOL T1***	−.083	.016

* *p* < .05; ** *p* < .01; *** *p* < .001

^a^Male is the reference category

## Discussion

This study examined the effect of CSE perceptions on initial PTSD levels and change in PTSD levels over time while taking into consideration the effects of demographics, injury severity, coping styles and changing HRQOL. CSE was by far the strongest predictor of initial PTSD symptoms and its course over the 12-month period, with higher CSE levels associated with lower initial symptoms and a steeper decline of symptoms over time. Only avoidant coping was also independently associated with initial symptom levels, and only emotional expression was also associated with rate of recovery. This emphasizes the central position of CSE in determining recovery from burn injuries, concurring with earlier research in other populations recovering from trauma (Luszczynska et al., 2009). Findings move beyond earlier studies by demonstrating the role of CSE in a trauma population that may have to deal with long-term pain and physical impairments such as reduced mobility and difficulties in self-care and usual activities that were taken into account in this study (Table 4).

**Table 4:** Correlation matrix

	1	2	3	4	5	6	7	8	9	10	11	12	13	14
1. Age	1													
2. Sex^a^	.011	1												
3. Surgeries	.184*	.117	1											
4. CSE	−.050	−.120	−.245**	1										
5. Emotional expression	.080	−.025	.095	.010	1									
6. Seeking support	−.140	.190*	.081	.138	.023	1								
7. Active coping	.034	−.237**	.012	.286**	.122	.248**	1							
8. Avoidant coping	−.117	.257**	.237**	−.233**	−.062	.007	−.170*	1						
9. HRQOL T1	.011	− .127	−.408**	.382**	.033	−.188*	.053	−.138	1					
10. HRQOL T2	−.016	−.139	−.245**	.288**	−.020	.077	.062	−.029	.349**	1				
11. HRQOL T3	.081	−.128	−.171*	.496**	−.038	.098	.180*	−.104	.288**	.667**	1			
12. PTSS T1	−.063	.129	.167*	−.644**	.036	−.043	−.212**	.298**	−.257**	−.279**	−.319**	1		
13. PTSS T2	−.159	.144	.143	−.407**	.075	−.018	−.204*	.093	−.262**	−.464**	−.501**	.601**	1	
14. PTSS T3	−.132	.140	.085	−.476**	.171*	−.055	−.201*	.205*	−.147	−.477**	−.692**	.509**	.774**	1

* Correlation is significant at the 0.05 level (2-tailed)

** Correlation is significant at the 0.01 level (2-tailed)

^a^Male is the reference category

Findings showed that trait coping styles played a role in explaining PTSD symptoms. Avoidant coping was negatively associated with initial PTSD levels, but not with rate of recovery, indicating that those with high levels of this coping style had more PTSD-symptoms in-hospital but demonstrated the same gradient of recovery. These findings are in line with earlier longitudinal burn studies that identified avoidant coping as a predictor in the post burn recovery phase (Fauerbach et al., 2002; Willebrand et al., 2004). Compared to the role of CSE in the recovery from burn injuries, however, the effect was minor. One may argue that an avoidant coping style might overlap with the PTSD symptom cluster avoidance and therefore could explain only a modest part of the variation in PTSD symptoms. Theoretically, the two are distinct: the coping style is measured as a general trait (with items such as: In general, do you give into avoid difficult situations), while the symptom cluster is related to reminders of a specific traumatic event (with items such as: I tried to banish the burn event from my memory). The distinctiveness of general avoidant coping and trauma-related avoidance of reminders to the traumatic event was corroborated by a multicollinearity test in this study (r(179) = .26, *p* < .001). Additionally, covariances showed that higher levels of CSE are related to lower levels of avoidant coping. It is possible that interventions aimed at enhancing CSE will also reduce the use of avoidant coping during post-burn recovery.

The coping style emotional expression on the other hand, was associated with a higher rate of recovery from PTSD-symptoms, but was not related to in-hospital PTSD-symptom levels. This finding supports previous evidence that suggests the beneficial role of emotional expression as a manner of repeated exposure and therefore facilitating habituation (Ehlers et al., 2006). In an integrative review on patients’ experiences, it was concluded that among other types of support, peer support was important as expressing emotions and sharing feelings with other burn survivors has been found beneficial (Kornhaber et al., 2014). Interestingly, the effect of emotional expression appears to work independently of other predictors in the model: it was not significantly related to CSE, other coping styles, degree of injury or any of the other variables in the model. It suggests that emotional expression might be a helpful coping strategy among burn patients, aiding their psychological recovery.

Burn severity was not a factor of significance in the recovery from PTSD symptoms as it did not affect either initial PTSD symptom levels, nor the rate of recovery. The impact of burn severity in psychological outcome studies is still subject of debate, with a number of studies finding an effect while others find no effect (Hobbs, 2014; Sareen et al., 2013). A possible explanation of the lack of an effect of burn severity in our study is that burn severity affected CSE levels, as illustrated by the significant association between CSE and number of surgeries, and that its effect on PTSS is indirect. Moreover, a significant correlation between HRQOL in-hospital and CSE demonstrates that physical disability and CSE perceptions might be related; HRQOL at that time is largely determined by impairments in physical domains because hospitalized burn patients are constrained in their movements and daily activities and experience significant pain. This reasoning is supported by the fact that HRQOL levels at T1 were no longer significantly associated with PTSD levels when the other predictors were added to the model. This suggests that the influence of early appraisals about the (physical) consequences of the burn event on PTSD symptoms might largely work through impacting initial CSE levels. Later in time, HRQOL did have an effect on PTSD levels. This suggests that beyond the immediate post-burn phase, the interaction between HRQOL and PTSD becomes more prominent. This is in line with findings by Van Loey et al. (2012) who found that PTSD was not significantly associated with initial HRQOL levels, but those with high symptom levels gained less HRQOL. More research is needed to understand the underlying mechanisms between degree of physical impairment and CSE in physically impaired populations.

Some limitations should be mentioned. Symptoms of PTSD were measured using self-rating scales. We did not use clinical diagnostic interviews like the Clinician-Administered PTSD Scale (CAPS, Blake et al., 1990) to assess PTSD. Nevertheless, the IES-R has been shown to be a valid instrument to screen for PTSD in burn populations (Sveen et al., 2010). Furthermore, patients received pain medication and may have received anxiolytics as is usual in burn care (Summer et al., 2007). In that way, patients with burns may differ from other trauma samples in the strategy used to deal with trauma affecting the generalization of the findings but there is no reason to assume this sample differs from other burn samples. Attrition may also have caused some bias. Of the 178 original study participants, 48 did not complete all three measurements. However, since those who completed all three measurements and drop-outs only differed significantly on the variables age and seeking social support (neither of which was related to initial symptom levels or rate of recovery), we may assume data was missing at random (MAR). The maximum likelihood estimation method used in this study is robust to data MAR.

Implications from these results are that when trying to predict psychological recovery among burn survivors, it is essential to take CSE perceptions into account in addition to the physical impact of the event, as the latter may affect psychopathology indirectly through CSE. Speed of psychological recovery among burn survivors may be increased by interventions targeting survivors’ CSE. Therefore, early recognition of low CSE levels seems imperative. Improving CSE in an early stage during recovery, for instance by addressing dysfunctional beliefs about the long-term physical and functional problems, or providing a level of control during painful treatment might help psychological recovery. Considering findings with regard to avoidant coping, increasing CSE perceptions might have the additional effect that the use of avoidant coping decreases. Stimulating emotional expression as a coping mechanism might also help burn survivors in adapting to their trauma in the longer term, and offers a separate target for intervention. However, the issue of early intervention in burn populations is complicated by the immediate inflammatory response affecting inflammatory mediators and stress hormone levels that have been associated with depression and PTSS (Sareen et al., 2013; Van Zuiden et al., 2011). Therefore, some caution may be relevant in employing early interventions in the context of severe burn trauma.

While trait coping styles had a limited impact relative to CSE on psychological recovery from burn injuries, future research measuring coping strategies used during recovery might offer more insights on how CSE interacts with actual coping behavior during recovery in this population. In order to better understand the long term role of CSE perceptions among burn survivors, additional research examining long term changes in CSE and its effect on psychopathology is needed. Finally, associations between the number of surgeries at admission and CSE indicate that the physical impact of a burn injury might affect CSE. Further study is needed to understand the mechanisms at work behind this association, and how developments in physical limitations over time and CSE interact.

Current findings suggest that CSE plays a pivotal role in the post-burn adjustment process, even when the role of the often substantial burden of a burn injury on HRQOL is taken into consideration. Although more research is needed to investigate the role of CSE and actual coping strategies used during recovery, (early) interventions aimed at increasing the sense of control during treatment and rehabilitation might stimulate psychological recovery.

## Footnotes

1 Tests of significance of paths in the model were 2-sided.

2 Tests of significance of paths in the model were 2-sided.
